# A Joint Model of Random Forest and Artificial Neural Network for the Diagnosis of Endometriosis

**DOI:** 10.3389/fgene.2022.848116

**Published:** 2022-03-08

**Authors:** Jiajie She, Danna Su, Ruiying Diao, Liping Wang

**Affiliations:** ^1^ Reproductive Medicine Centre, Shenzhen Second People’s Hospital, The First Affiliated Hospital of Shenzhen University, Shenzhen, China; ^2^ Shenzhen Institutes of Advanced Technology, Chinese Academy of Sciences, Shenzhen, China

**Keywords:** endometriosis, random forest, artificial neural network, diagnostic model, diagnostic efficacy

## Abstract

Endometriosis (EM), an estrogen-dependent inflammatory disease with unknown etiology, affects thousands of childbearing-age couples, and its early diagnosis is still very difficult. With the rapid development of sequencing technology in recent years, the accumulation of many sequencing data makes it possible to screen important diagnostic biomarkers from some EM-related genes. In this study, we utilized public datasets in the Gene Expression Omnibus (GEO) and Array-Express database and identified seven important differentially expressed genes (DEGs) (*COMT*, *NAA16*, *CCDC22*, *EIF3E*, *AHI1*, *DMXL2*, and *CISD3*) through the random forest classifier. Among these DEGs, *AHI1*, *DMXL2*, and *CISD3* have never been reported to be associated with the pathogenesis of EMs. Our study indicated that these three genes might participate in the pathogenesis of EMs through oxidative stress, epithelial–mesenchymal transition (EMT) with the activation of the Notch signaling pathway, and mitochondrial homeostasis, respectively. Then, we put these seven DEGs into an artificial neural network to construct a novel diagnostic model for EMs and verified its diagnostic efficacy in two public datasets. Furthermore, these seven DEGs were included in 15 hub genes identified from the constructed protein–protein interaction (PPI) network, which confirmed the reliability of the diagnostic model. We hope the diagnostic model can provide novel sights into the understanding of the pathogenesis of EMs and contribute to the clinical diagnosis and treatment of EMs.

## Introduction

Endometriosis (EM) is an estrogen-dependent inflammatory disorder, which afflicts about 10%–15% of women of childbearing age (Parasar et al., 2017). It is defined as the presence of endometrial-like tissue outside of the uterine cavity, which can lead to chronic pelvic pain, and infertility (Drabble et al., 2021). However, the true prevalence of EMs is uncertain as visual laparoscopy is the gold standard for the diagnosis of EMs (Taylor et al., 2018). At the moment, Sampson’s theory of menstrual blood reflux observed in most patients is commonly accepted in the pathophysiology of EMs, while only a small portion will develop into this disease (Burney and Giudice, 2012). However, it could only explain a portion of EMs. Therefore, it’s necessary to further investigate a comprehensive understanding of the pathogenesis of EMs and find effective molecular biomarkers to improve the early diagnosis and treatment of EMs.

DNA microarray technology is a high-throughput detection method that can be used to provide gene expression profiles and thus can help to screen disease-related genes and biomarkers (Yoo et al., 2009). With the rapid development of DNA microarray technology, a large amount of high-throughput data has accumulated available on public platforms. However, how to make effective use of these data to screen critical disease-related genes for the diagnosis of EMs is a great challenge. At present, random forest and neural network are widely applied for disease prediction (Yigit and Isik, 2018; Khan et al., 2019; Shaia et al., 2019; Kugunavar and Prabhakar, 2021). Among them, random forest algorithm can perform random sampling to screen the target variables (Schonlau and Zou, 2020) and has high predicted accuracy (Byeon, 2019; Chen et al., 2020). Furthermore, the artificial neural network can be used to evaluate the accuracy of predicted model with divided training and validation datasets (Curchoe et al., 2020). Currently, there are some useful visualization and analysis tools for neural networks, such as NeuralNetTools (Beck, 2018), spiking neuronal networks (Galindo et al., 2020), and Net2Vis (Bauerle et al., 2021).

**FIGURE 1:**
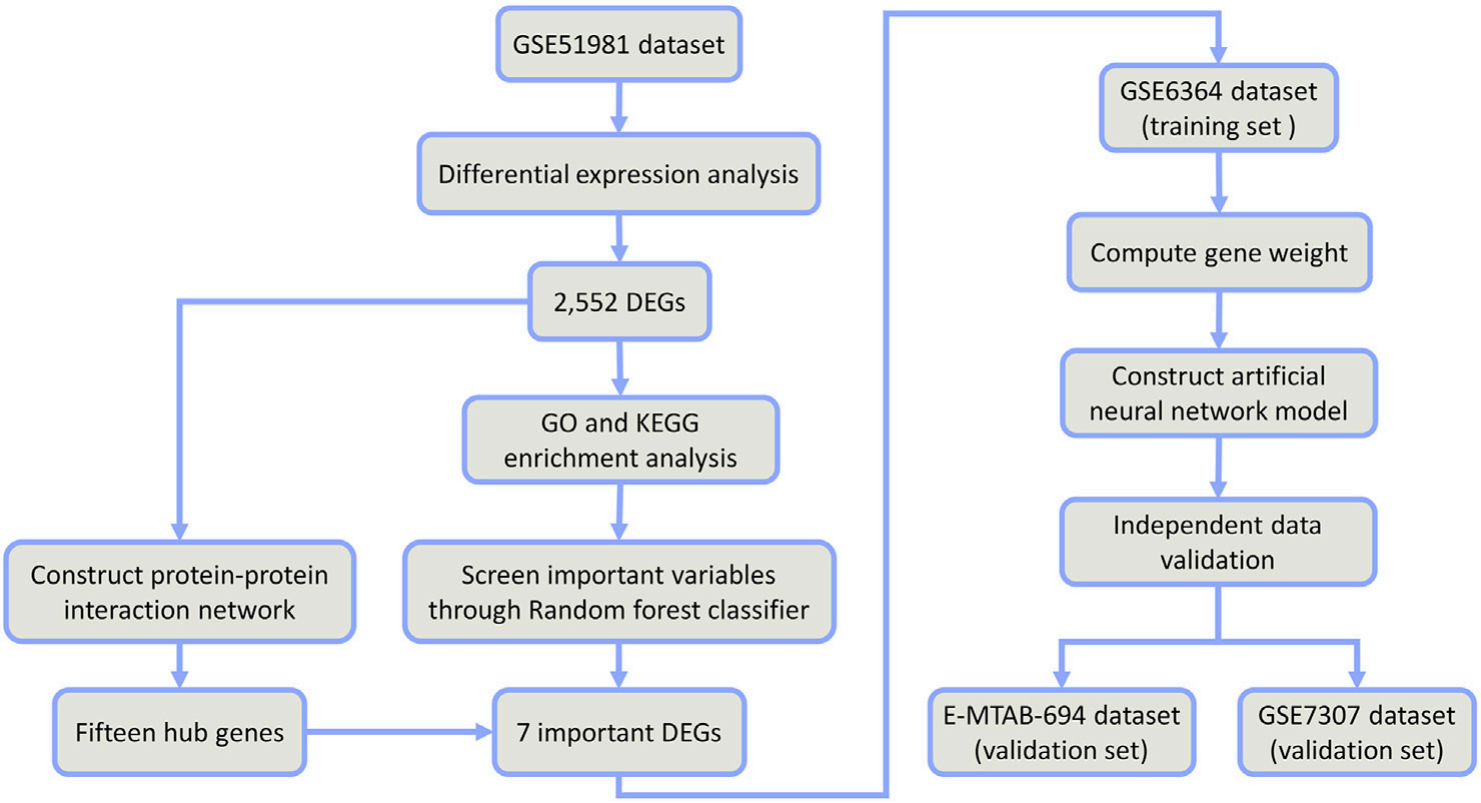
Flow chart.

Therefore, the combination of random forest and artificial neural network would have better classification performance and more meaningful selected features (Kong and Yu, 2018; Tian et al., 2020). In this study, we firstly identified some differentially expressed genes (DEGs) between EMs and normal samples from public datasets in the Gene Expression Omnibus (GEO) database. Through the random forest classifier, we screened these DEGs and obtained seven important DEGs (*COMT*, *NAA16*, *CCDC22*, *EIF3E*, *AHI1*, *DMXL2*, and *CISD3*). Then, we put these seven DEGs into an artificial neural network to construct a novel diagnostic model and verified its diagnostic efficacy in two public datasets (See the detailed process in Figure 1). We hope this diagnostic model can provide novel sights into the pathogenesis of EMs and improve the early diagnosis and treatment of EMs.

## Materials and Methods

### Data Download and Processing

The GSE51981, GSE6364, and GSE7307 datasets were downloaded by the R package “GEOquery” (2.60.0) (Davis and Meltzer, 2007) to obtain the expression profile data. Then, the E-MTAB-694 dataset was downloaded through the Array-Express database. The related annotation information including the platforms, the probes, and ID conversion was obtained from the GEO database. When multiple probes corresponded to one gene symbol, the average expression level of multiple probes was used as the expression level of the corresponding gene. ID conversion was conducted with the R package “org.Hs.eg.db” (v3.13.0). Furthermore, the “removeBatchEffect” function in the R package “LIMMA” (v3.48.3) (Ritchie et al., 2015) was used to adjust batch effects, which were evaluated by principal component analysis (PCA).

### Differential Expression and Functional Enrichment Analysis

Differential expression analysis was conducted on 77 EM disease and 71 normal samples of the GSE51981 dataset through the Bayesian analysis of the R package “LIMMA”. The log2FoldChange > 1.5 and *p*-value < 0.05 were set as the threshold of DEGs. The R package “pheatmap” (v1.0.12) was used to perform clustering analysis of DEGs for the heatmap. To explore the biological significance of these DEGs in the pathogenesis of EMs, GO and KEGG pathway enrichment analyses were performed through the R package “clusterProfiler” (v4.1.3) (Wu et al., 2021) to identify significantly enriched GO terms and significantly enriched KEGG pathways with the threshold of *p*-value < 0.05.

### The Construction of Hub Gene Network

The STRING (v11.5) (https://string-db.org/cgi/input.pl) (Szklarczyk et al., 2021) has been widely applied to construct a protein–protein interaction (PPI) network. Based on those DEGs, the “Multiple proteins” option was selected. In the PPI network, the minimum required interaction score was set as “high confidence (0.700)”. Then, the cytoHubba (Chin et al., 2014) was employed to identify hub genes. The eccentricity algorithm was selected and 15 top-ranked genes were chosen as hub genes. Finally, the hub gene network was visualized with Cytoscape (v3.9.0) (Demchak et al., 2014).

### Screening Differentially Expressed Genes With the Random Forest Model

The R package “randomForest” (v4.6.14) (Liaw et al., 2014) was used to construct a random forest model to screen DEGs. The number of random seeds and decision trees was set as 1–5,000 and 3,000 in the random forest classifier originally, respectively. Finally, the number of random seeds and decision trees was set as 4,543 and 219, respectively, which represented higher accuracy of the constructed model and stable model error. The Gini coefficient method was used to obtain the dimensional importance value of all variables from the constructed random forest model. Those DEGs with an importance value greater than 4 were screened as important genes of EMs for subsequent model construction and verification. The R package “pheatmap” was used to perform clustering analysis of the screened important genes for the heatmap in this dataset.

### The Construction and Verification of the Artificial Neural Network Model

The GSE6364 dataset downloaded through the R package “GEOquery” was selected as the training set for the construction of the artificial neural network model. After the data normalization, the R package “neuralnet” (v1.44.2) (Fritsch and Guenther, 2016) was used to construct an artificial neural network model of those important variables. The number of hidden neuron layers should be two-thirds of the number of the input layer plus two-thirds of the number of the output layer. Therefore, six hidden layers were set as the model parameter to construct a classification model of EMs through the predicted gene weight information. The R packages “pROC” (v1.18.0) (Robin et al., 2011) and “ggplot2” (v3.3.5) (Gómez-Rubio, 2017) were used to calculate the verification results of AUC classification performance and draw the ROC curve. Another two datasets E-MTAB-694 and GSE7307 were used to verify the accuracy of the constructed neural network model for the diagnosis of EMs. The R package “pROC” was used to draw ROC curves for each dataset, and the AUC value was calculated to verify the classification efficiency. Meanwhile, the sensitivity and specificity in distinguishing the disease samples from normal samples were calculated.

## Results

### Data Processing and Differential Expression Analysis

**FIGURE 2:**
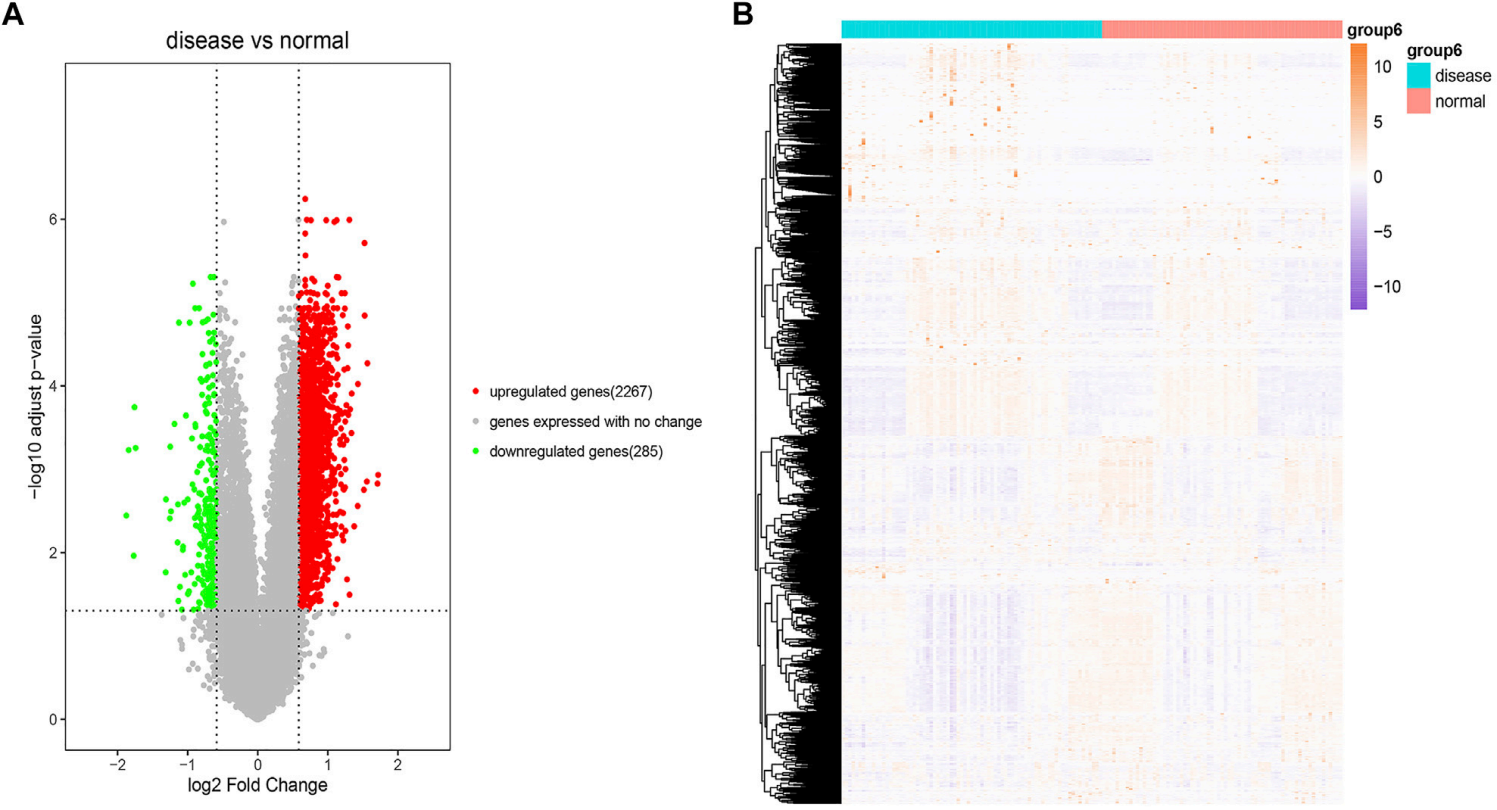
Differential expression analysis. **(A)** Volcano plot of the result of differential expression analysis. The *x*-axis is log2 (fold change) and the *y*-axis is −log10 (adjusted *p*-value). The red dots represent significant upregulated expressed genes. The green dots represent significant downregulated expressed genes. The gray dots represent genes expressed with no change. **(B)** Heatmap of these DEGs. The colors in the graph from red to pink indicate the change from high to low expression levels. On the upper part of the heatmap, the blue band indicates the disease samples and the red band indicates the normal samples.

The R package “GEOquery” was used to download the GEO dataset GSE51981 (77 EM disease samples and 71 normal samples) and obtain detailed information. We used the “removeBatchEffect” function in the R package “LIMMA” to adjust batch effects and then conducted principal component analysis (PCA) analysis to evaluate the performance of batch effect adjustment. PCA results **(** Supplementary Figure S1) indicated that the disease samples were mixed with the normal samples, which suggested the challenge of diagnosing. We also used the R package “LIMMA” to perform differential expression analysis for the dataset GSE51981 through the Bayesian test. We finally identified 2,267 significantly upregulated and 285 significantly downregulated expressed genes between the disease samples and the normal samples with the threshold of fold change values of >1.5 and *p* < 0.05. The detailed information of all DEGs is listed in Supplementary Table S1. The results of these DEGs and the heatmap of these DEGs are visualized in Figures 2A and 2B, respectively.

### Functional Enrichment Analysis for DEGs and the Construction of PPI Network

**FIGURE 3:**
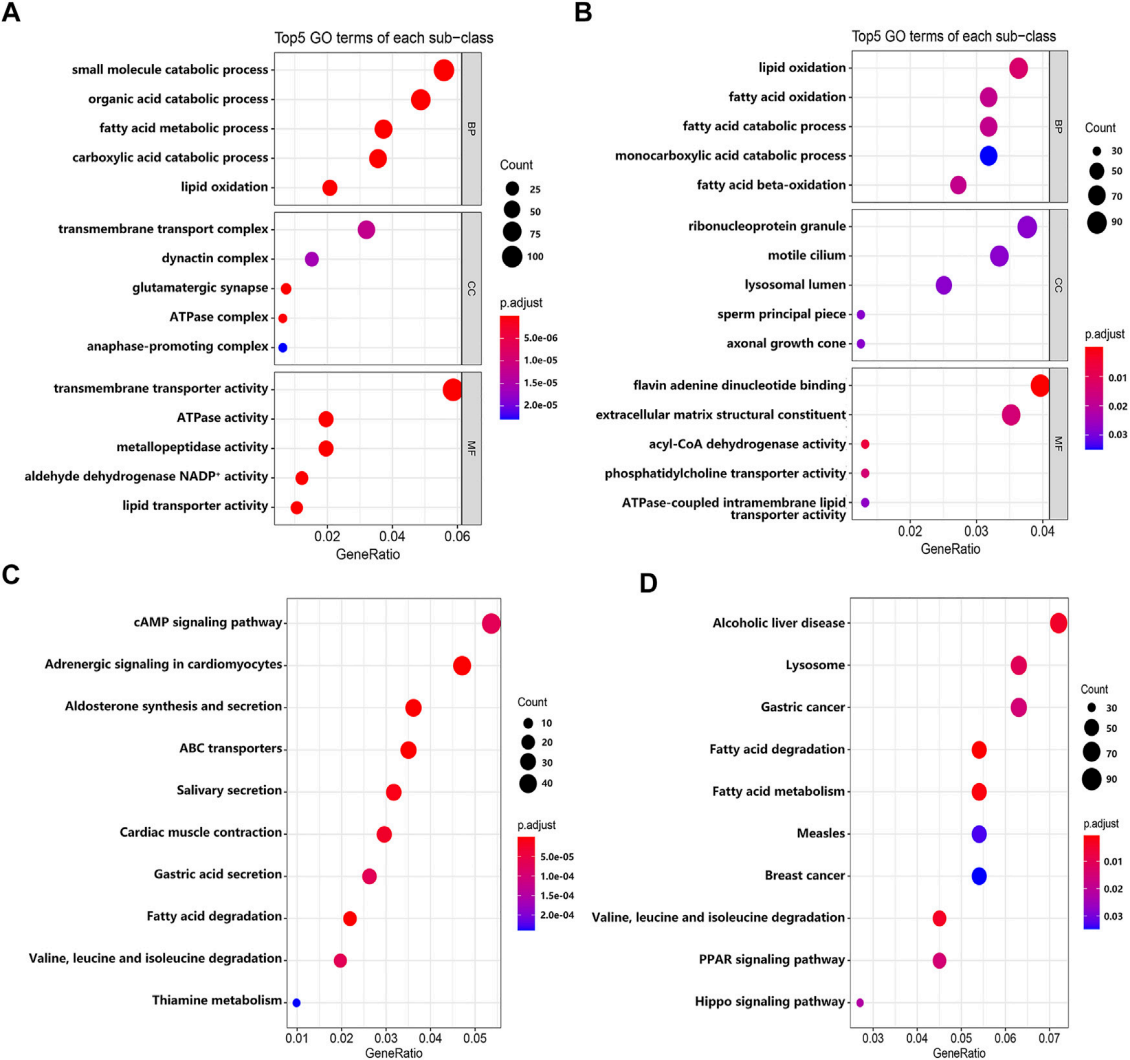
The results of GO and KEGG enrichment analyses. **(A)** The top five GO terms of genes with significantly upregulated expressed level. **(B)** The top five GO terms of genes with significantly downregulated expressed level. **(C)** The top 10 KEGG pathways of genes with significantly upregulated expressed level. **(D)** The top 10 KEGG pathways of genes with significantly downregulated expressed level.

To explore the biological significance of these DEGs in the pathogenesis of EMs, we performed GO and KEGG pathway enrichment analyses through the R package ‘clusterProfiler’. GO terms were classified into three categories: biological process (BP), cellular component (CC), and molecular function (MF). The top five GO terms of genes with significantly upregulated and downregulated expression levels were visualized in Figures 3A,B. The GO enrichment analysis results indicated that these significantly upregulated expressed genes were mainly involved in the transmembrane transporter activity, ATPase activity, metallopeptidase activity, aldehyde dehydrogenase NADP^+^ activity, and lipid transporter activity (Supplementary Table S2), while these significantly downregulated expressed genes were mainly involved in the flavin adenine dinucleotide binding, acyl-CoA dehydrogenase activity, phosphatidylcholine transporter activity, extracellular matrix structural constituent, and ATPase-coupled intramembrane lipid transporter activity (Supplementary Table S3). For KEGG pathway enrichment analysis, the results indicated that these upregulated expressed genes were significantly associated with the cAMP signaling pathway, adrenergic signaling in cardiomyocytes, aldosterone synthesis and secretion, ABC transporters, and salivary secretion (Supplementary Table S4), while these downregulated expressed genes were significantly associated with fatty acid degradation and metabolism; valine, leucine, and isoleucine degradation; lysosome; the PPAR signaling pathway; and the Hippo signaling pathway (Supplementary Table S5). Furthermore, we constructed a PPI network through the STRING database. The hub genes selected from the PPI network are shown in Supplementary Figure S2. According to the eccentricity scores, we identified 15 hub genes from the network, which had highest confidence scores.

### Constructing the Random Forest Model to Screen Differentially Expressed Genes

**FIGURE 4:**
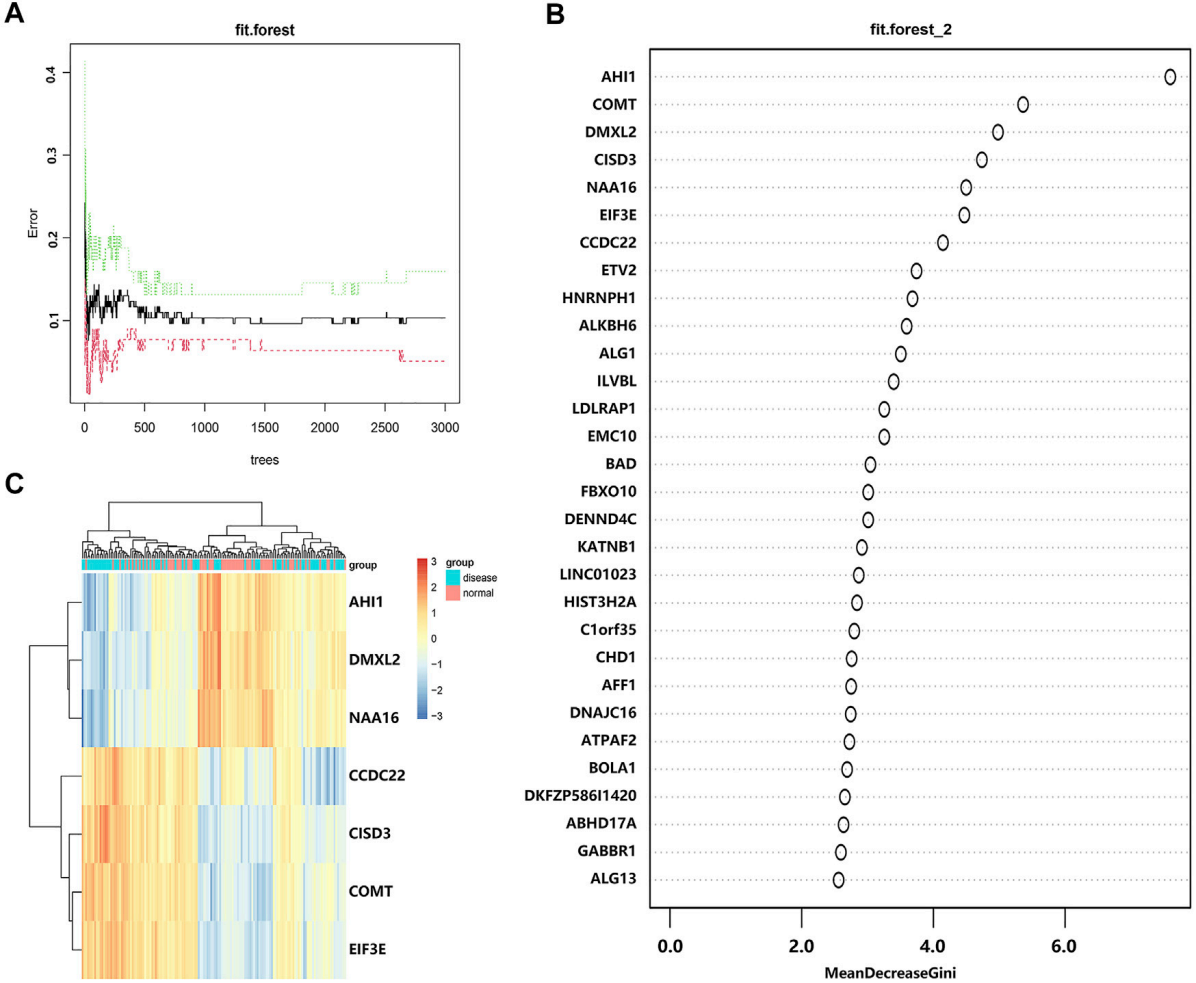
Screening DEGs with the random forest model. **(A)** The relationship between the number of decision tree and the model error. The *x*-axis represents the number of decision trees, and the *y*-axis represents the error rate of the constructed model. When the number of decision trees is nearly 219, the error rate of the constructed model is relatively stable. **(B)** The importance of all variables in the random forest classifier through the Gini coefficient method. The *x*-axis represents the mean decrease of the Gini index, and the *y*-axis represents all variables. **(C)** The heatmap of *k*-means clustering in the GSE6364 dataset. The colors in the graph from red to blue indicate the change from high to low in expression level. On the upper part of the heatmap, the blue band indicates the disease samples and the red band indicates the normal samples.

To screen DEGs, we put these DEGs into the random forest classifier and set the number of random seeds to 4,543. By referring to the relationship between the model error and the number of decision trees (Figure 4A), we selected 219 trees as the parameter of the random forest model, which represented a stable error in the model. In the modeling process, we used the Gini coefficient method to measure the importance of all variables according to decreased mean square error and model accuracy (Figure 4B). Finally, we selected seven DEGs (*AHI1*, *DMXL2*, *NAA16*, *CCDC22*, *CISD3*, *COMT*, and *EIF3E*) with a mean decrease of Gini index greater than 4 as important variables for subsequent analysis. Interestingly, all these DEGs were included in the 15 hub genes identified from the constructed PPI network. Among these variables, *AHI1* was the most important, with the mean decrease of the Gini index being much higher than other variables (Supplementary Table S6). A small number of variables meant a small out-of-band error, which represented a high accuracy of the constructed random forest model. Based on these seven variables, we performed the *k*-means clustering of the dataset. The results suggested that these seven genes could be used to distinguish the disease sample from the normal samples (Figure 4C). Furthermore, *AHI1*, *DMXL2*, and *NAA16* genes were clustered as a group with low expression in the normal sample and high expression in the disease sample. On the contrary, *CCDC22*, *CISD3*, *COMT*, and *EIF3E* were clustered as another group with high expression in the normal sample and low expression in the disease sample.

### The Construction of the Artificial Neural Network Model and the Evaluation of the ROC Curve

**FIGURE 5:**
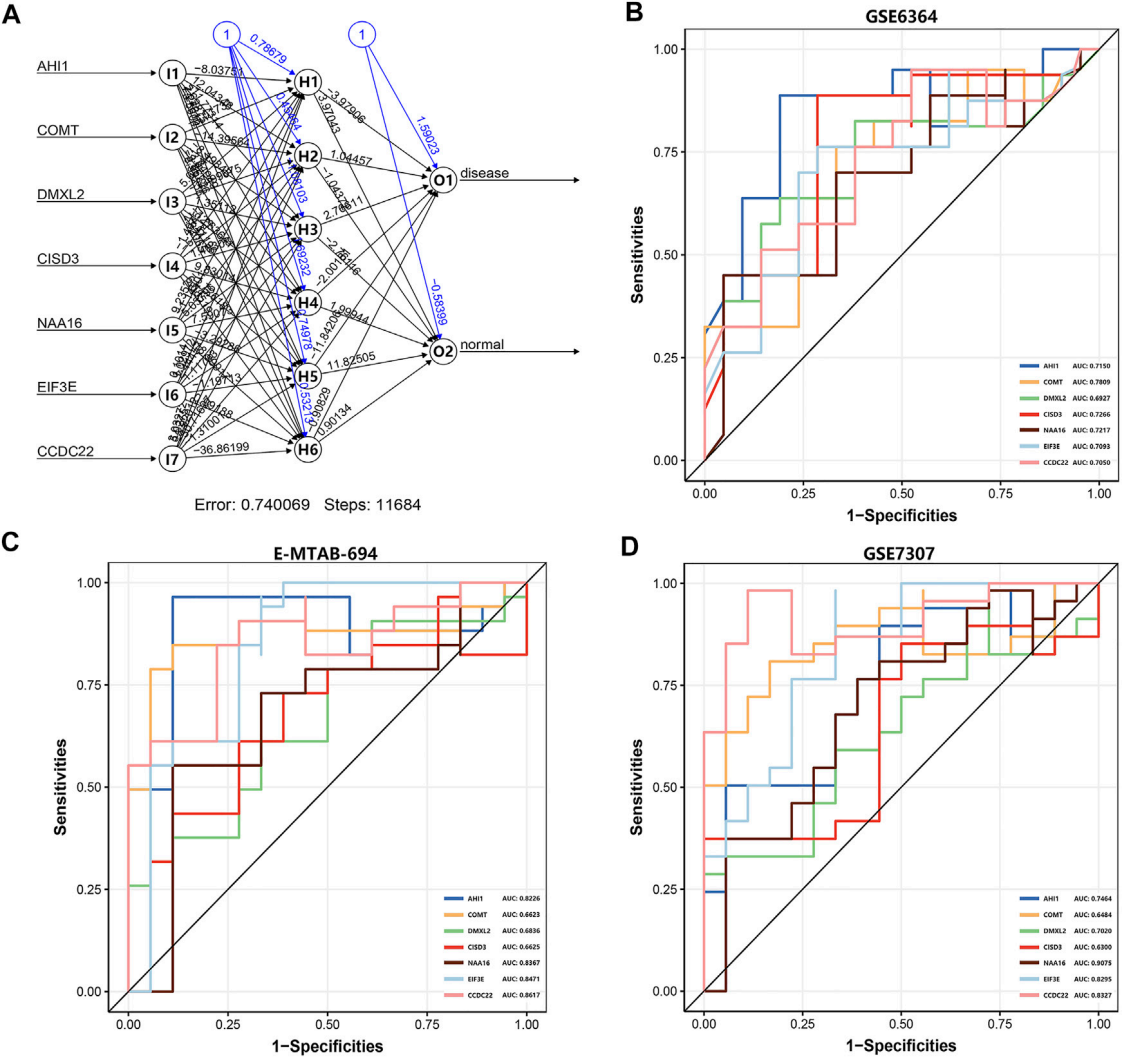
The artificial neural network model and the evaluation of the ROC curve. **(A)** The visualization of the artificial neural network model. **(B)** The evaluation results of the ROC curve in the GSE6364 dataset. **(C)** The verification results of the ROC curve in the E-MTAB-694 dataset. **(D)** The verification results of the ROC curve in the GSE7307 dataset. The *x*-axis and *y*-axis represent specificity and sensitivity, respectively. The AUC value is the area under the ROC curve.

Based on the R package ‘neuralnet’, we use the GSE6364 dataset (21 disease samples and 21 normal samples) as the training set to construct the artificial neural network model. Firstly, we performed the preprocessing and normalization of this dataset. According to the output results of the neural network model (Figure 5A), it is illuminated that the entire training was performed in 11,684 steps. Among the output results, the predicted weights of each hidden neuron layer were −3.97906, 1.04457, 2.76611, −2.00181, −11.84206, and −0.90829 (Supplementary Table S7). Next, we drew the ROC curve to evaluate the predicted performance; the AUC values of *AHI1*, *COMT*, *DMXL2*, *CISD3*, *NAA16*, *EIF3E*, and *CCDC22* were 0.7150, 0.7809, 0.6927, 0.7266, 0.7217, 0.7093, and 0.7050, respectively (Figure 5B). The larger the AUC value of each DEG is, the higher the credibility of the constructed diagnostic model will be. We also used another two datasets E-MTAB-694 (18 disease samples and 17 normal samples) and GSE7307 (18 disease samples and 23 normal samples) to verify the accuracy of the constructed neural network model. In the E-MTAB-694 dataset (Figure 5C), the AUC values of the seven DEGs were 0.8226, 0.6623, 0.6836, 0.6625, 0.8367, 0.8471, and 0.8617. In the verification results of the GSE7307 dataset (Figure 5D), the AUC values of the seven DEGs were 0.7464, 0.6484, 0.7020, 0.6300, 0.9075, 0.8295, and 0.8327. In general, we constructed a novel diagnostic model of EMs and verified its diagnostic efficacy through the constructed artificial neural network in two public datasets.

## Discussion

The combination of random forest and artificial neural network can be used to construct a reliable predictive model for the diagnosis of some diseases, such as polycystic ovary syndrome (PCOS) (Xie et al., 2020) and ulcerative colitis (Li et al., 2020). In this study, we identified 2,552 DEGs associated with EMs in the GSE51981 dataset. Based on the random forest classifier, seven important candidate DEGs (*COMT*, *NAA16*, *CCDC22*, *EIF3E*, *AHI1*, *DMXL2*, and *CISD3*) were screened. Then, we used the GSE6364 dataset as the training set to construct the artificial neural network model and evaluated the classification efficacy of the model in E-MTAB-694 and GSE7307 datasets. The AUC values of the ROC curve were about 0.7, which had great efficiency and verified the diagnostic efficacy of the model. Furthermore, we constructed a 15-hub-gene-based PPI network and confirmed the reliability of the prediction model. Compared with the Nnet package, we found that the neuralnet package had higher accuracy of the predicted model (86.5% vs 81.1%). In total, the constructed diagnostic model could provide new insight into our understanding of the pathogenesis of EMs and identify crucial biomarkers as diagnostic and therapeutic targets of EMs.

Among these seven genes, *COMT*, *NAA16*, *CCDC22*, and *EIF3E* have been reported to be associated with the pathogenesis of EMs. Catechol-*O*-methyltransferase (*COMT*) is highly expressed in the placental, adrenal gland, ovary, and other tissues. The degradative pathways of the catecholamine transmitters can relieve painful uterine contractions (D’Astous-Gauthier et al., 2021). *COMT* polymorphism may contribute to the risk of EMs and adenomyosis (Li et al., 2018) and has a relationship with EM susceptibility (Ji et al., 2017; Zhai et al., 2019). *N*-alpha-acetyltransferase 16 (*NAA16*) is highly enriched in bone marrow, testis, endometrium, and other tissues. It can alter NAT 2 enzyme activity and thus contribute to the susceptibility of EMs (Nakago et al., 2001). Coiled-coil domain containing 22 (*CCDC22*), a membrane-binding protein, is highly enriched in the spleen, lymph node, and other tissues. Studies have demonstrated that there is also a relationship between *CCDC22* polymorphisms and EM susceptibility (de Oliveira Francisco et al., 2017). Eukaryotic translation initiation factor 3 subunit E (*EIF3E*) is highly expressed in the ovary, lymph node, endometrium, and other tissues. Its downregulation may be involved in epithelial–mesenchymal transition (EMT) in EMs, possibly through the preferential translation of snail (an inhibitor of E-cadherin) (Cai et al., 2018) and involved in the development of adenomyosis through activating the TGF-β1 signaling pathway (Cai et al., 2019).

Interestingly, we identified another three important genes (*AHI1*, *DMXL2*, and *CISD3*), which have never been reported to be involved in the pathogenesis of EMs. Abelson helper integration site 1 (*AHI1*) is highly enriched in testis, adrenal gland, brain, prostate, endometrium, and other tissues, which has upregulated expression level in EMs. The *AHI1* protein participates in reactive oxygen species (ROS) production in the form of protein complexes (Liu et al., 2017). Excessive production of ROS can result in oxidative stress (OS) and overall immune activation and inflammation (Newsholme et al., 2016). OS represents an imbalance between ROS and antioxidants, which may have an essential role in the endometriosis pathogenesis in the peritoneal cavity (Samimi et al., 2019). Hence, the *AHI1* protein may participate in the EMs pathogenesis through multiple processes such as OS and immune and inflammatory response.

Dmx like 2 (*DMXL2*) encodes a protein with 12 WD domains, which has relatively low expression in endometrium tissue and downregulated expression in EMs. The *DMXL2* protein is demonstrated to participate in the regulation of the Notch signaling pathway (Sethi et al., 2010) and acts as a transmembrane protein, which can promote EMT through hyperactivation of the Notch signaling pathway (Faronato et al., 2015). Interestingly, decreased Notch signaling can contribute to impaired decidualization through the downregulation of FOXO1 (a downstream target of Notch signaling) and thus lead to the pathogenesis of EMs (Su et al., 2015). Furthermore, studies indicate that a circRNA with downregulated expression can regulate EMT in EMs via the Notch signaling pathway (Zhang et al., 2019). Therefore, the downregulated expression of *DMXL2* may activate the Notch signaling pathway, contribute to EMT through the interaction with circRNA, and thus lead to the pathogenesis of EMs.

CDGSH iron sulfur domain 3 (*CISD3*) is a member of the CDGSH domain-containing family, whose expression is upregulated in EMs. The *CISD3* protein is redox active and is thought to play an important role in mitochondrial homeostasis (Geldenhuys et al., 2019). Studies indicate that mitochondrial homeostasis can be considered as the therapeutic target for the treatment of EMs via limiting ESC migration and promoting apoptosis (Suliman and Piantadosi, 2016; Zhao et al., 2018). Furthermore, excessive mitochondrial fission can initiate caspase 9-related mitochondrial apoptosis and thus lead to cell death (Fuhrmann and Brüne, 2017; Zhou et al., 2017). Therefore, upregulated expression of *CISD3* may affect mitochondrial homeostasis and thus play an important role in the pathogenesis of EMs.

In this study, based on random forest and artificial neural network algorithm, we established a novel reliable diagnostic model and screened out three important DEGs that have never been reported to be involved in the pathogenesis of EMs. We aimed at the supplement of existing methods and provided an alternative marker panel for further research in the early screening of EMs. However, there are some limitations for this study. Firstly, all samples are only classified as EM (disease) and non-EM (normal) groups, which may affect the final screening results of DEGs. Secondly, the diagnostic model is only verified in two public datasets, which need more samples for verification. Thirdly, we conduct data analysis only at the mRNA level in the tissue samples of EMs, which require further validation at the mRNA and protein levels. In general, our approach has a certain clinical value, which can be beneficial for the early screening of EMs.

## Data Availability

The original contributions presented in the study are included in the article/supplementary material, further inquiries can be directed to the corresponding author/s.
