# Supplementary material for: A Joint Model of Random Forest and Artificial Neural Network for the Diagnosis of Endometriosis
Source: Front Genet. 2022 Mar 8;13:848116. doi: 10.3389/fgene.2022.848116 (PMC8957986; doi:10.3389/fgene.2022.848116)
Supplement: Supplementary file 8 [file DataSheet1.docx]

Supplementary Material

**A joint model of random forest and artificial neural network for the diagnosis of Endometriosis**

Jiajie She^1,2^, Danna Su^1^, Ruiying Diao^1*^, and Liping Wang^1*^

*To whom correspondence should be addressed.

***Correspondence:** Liping Wang: <wlp18665070696@163.com>; Ruiying Diao: <15889753127@163.com>

# Supplementary Figures and Tables

## Supplementary Figures


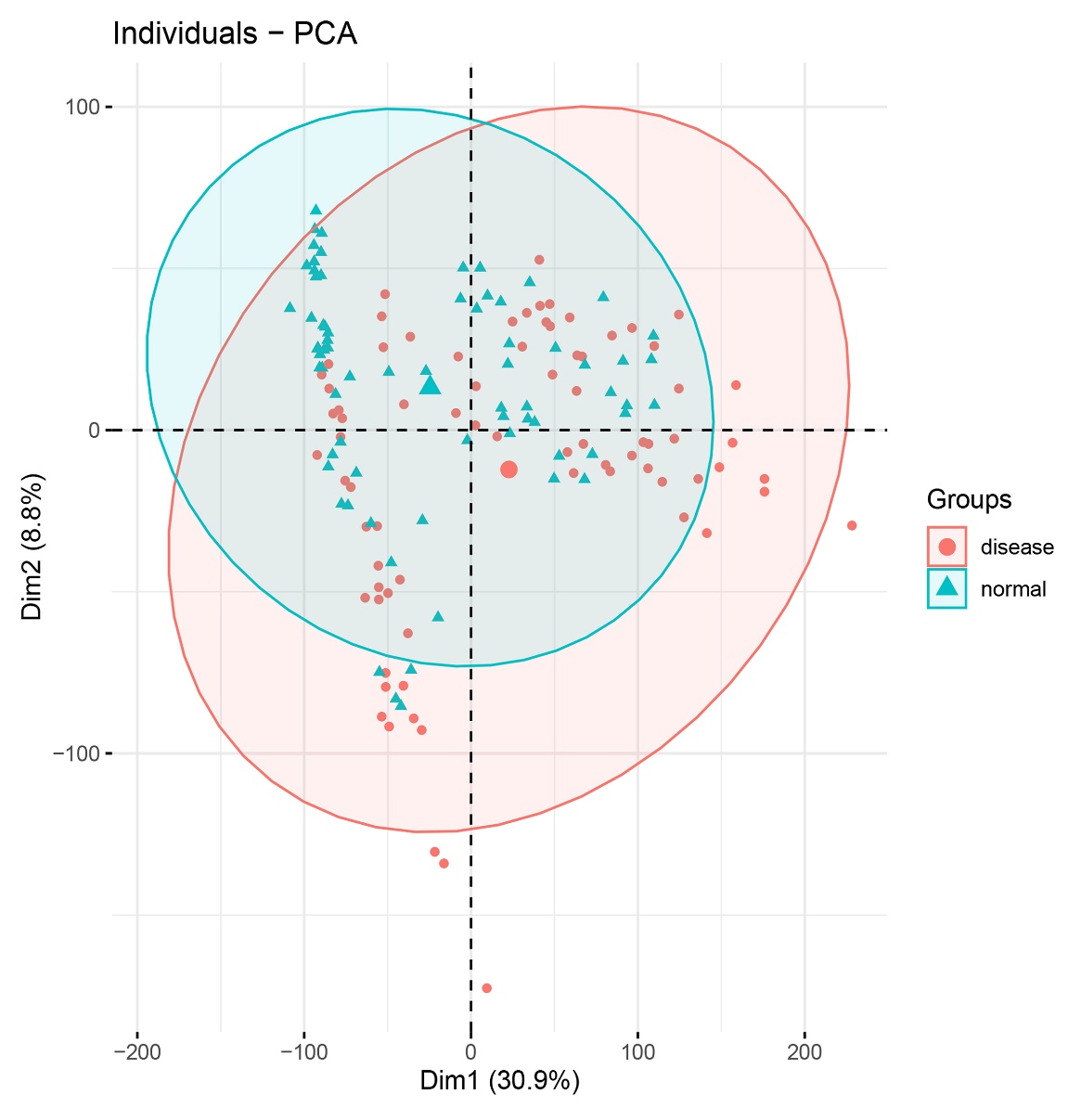


Supplementary Figure 1 PCA analysis of the GSE51981 datasets in EMs.


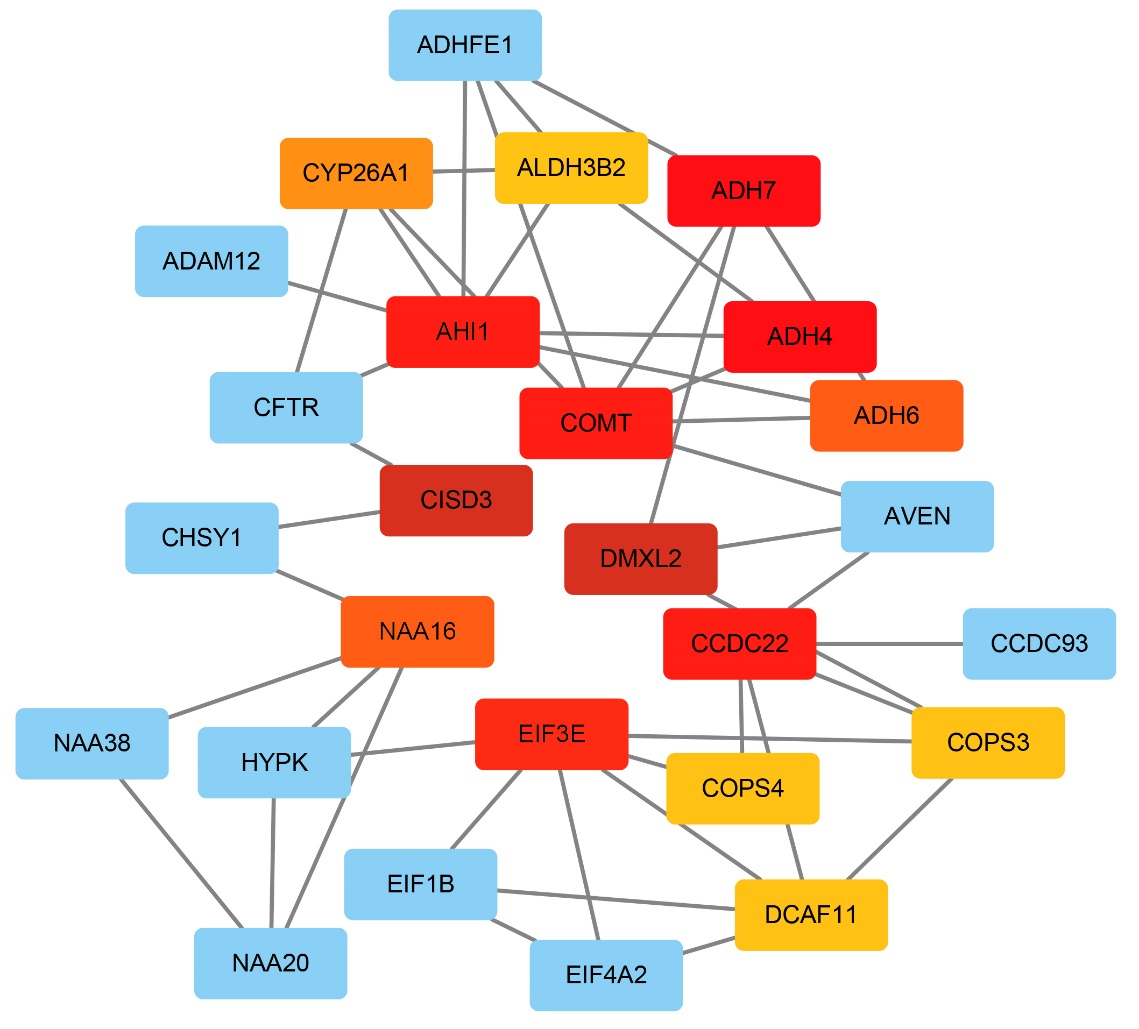


Supplementary Figure 2 Fifteen hub genes with the most substantial interactions according to the calculated results by eccentricity method. The darker the color of nodes, the higher the score.

## Supplementary Tables

Supplementary Table S1. 2,552 significant DEGs related to EMs. (Supplementary Table S1.xls)

Supplementary Table S2. The GO enrichment results of 2,267 upregulated expressed genes between disease and normal samples. (Supplementary Table S2.xls)

Supplementary Table S3. The GO enrichment results of 285 downregulated expressed genes between disease and normal samples. (Supplementary Table S3.xls)

Supplementary Table S4. The KEGG enrichment results of 2,267 upregulated expressed genes between disease and normal samples. (Supplementary Table S4.xls)

Supplementary Table S5. The KEGG enrichment results of 285 downregulated expressed genes between disease and normal samples. (Supplementary Table S5.xls)

Supplementary Table S6. The output results of the neural network model. (Supplementary Table S6.xls)

Supplementary Table S7. The mean decrease of Gini index of those 7 important DEGs. (Supplementary Table S7.xls)
